# TRAJECTORIES OF DISEASES, FUNCTIONAL LIMITATIONS, AND PSYCHOLOGICAL AND COGNITIVE HEALTH OF CENTENARIANS

**DOI:** 10.1093/geroni/igad104.0134

**Published:** 2023-12-21

**Authors:** Erfei Zhao, Jennifer Ailshire, Eileen Crimmins

**Affiliations:** University of Southern California, Leonard Davis School of Gerontology, Los Angeles, California, United States; University of Southern California, Los Angeles, California, United States; University of Southern California, Los Angeles, California, United States

## Abstract

Centenarians are recognized as providing a model of delayed aging. Centenarians are generally healthier throughout their 80s/90s than non-centenarians. However, the extent of the delay in morbidities experienced by centenarians remains unclear as it requires quantifying trajectories of health deterioration with age for both centenarians and their cohort counterparts who did not survive to 100-year-old. We use the Health and Retirement Study, a nationally representative sample, to assess the aging experience of centenarians and non-centenarians in the following domains: diseases, functional limitations, psychological and cognitive functioning. We include data from 1993 to 2018. We include 4,778 respondents born before 1918 as they had the potential to become centenarians during the study period - 201 reached at least 100-year-old and are categorized as centenarians; 4,577 of them died before 100. We use join point regression to determine 1) how many years centenarians were delayed in morbidities onset and 2) whether deterioration is accelerated starting at certain ages. On average, onset of any limitation in activities of daily living (ADL) among centenarians is delayed by about 10 years, instrumental ADLs by 9 years, and diseases by nearly 16 years (if hypertension is not considered). Rates of deterioration are slower, in general, for centenarians; however, starting at the age of 90, centenarians experience accelerated decline in ADL, IADL, functioning, and cognition. Centenarians do not appear to experience decline in psychological well-being whereas non-centenarians do. Our results suggest centenarians generally survive to older ages because they postpone aging; it is not avoidable indefinitely.

